# A Cohort Based Case Series: Learnings from an Iterative Group Therapy Model to Support Psilocybin-Assisted Therapy for Patients with a Terminal Diagnosis

**DOI:** 10.1192/j.eurpsy.2024.791

**Published:** 2024-08-27

**Authors:** V. Tsang

**Affiliations:** UBC, Vancouver, Canada

## Abstract

**Introduction:**

While much is known about psilocybin-assisted therapy for individuals, little is known about the experience of participants in a group psilocybin therapy model.

**Objectives:**

In an attempt to bridge this gap in the literature, a program development and quality improvement effort was launched.

**Methods:**

Thirty-one psychedelic-assisted therapy (PaT) sessions were provided for 25 participants within four iterative cohorts over the span of one year. This article reports participant feedback in an effort to inform the benefits and challenges of group-administered PaT.

**Results:**

Six to eight once-weekly group resilience-based community of practice (CoP) sessions were combined with one psilocybin-assisted therapy session for patients experiencing distress related to a terminal health condition. The virtual hybrid group therapy model is research informed, with a curriculum that provides knowledge-based content, combined with the relational elements necessary to successfully deliver group-administered psilocybin-assisted therapy.

Twenty one of the twenty five participants (84%) completed the program. Based on participant feedback, the following themes emerged: 1) Improvement of pre-treatment preparation sessions; 2) PaT Benefits: Gaining perspective, peace, non-attachment, authenticity, honesty, relational capacity; 3) The community of practice (CoP) as the primary conduit for connection and regulation 4) Population specific curriculum with a greater focus on how to navigate death, pain and loss; 5) PaT session Challenges; 6) The interpersonal and support capacity of the team as critical for the overall experience.

**Conclusions:**

While more research is needed, results suggest that psilocybin can be delivered safely in a group setting, and that a virtual CoP is effective across the spectrum of set, setting and integration Our findings also suggest that there is much to learn - and improve upon - in this novel area of service delivery.

**Disclosure of Interest:**

None Declared

